# Association Between Family Income, Subclinical Myocardial Injury, and Cardiovascular Mortality in the General Population

**DOI:** 10.1002/clc.70036

**Published:** 2024-10-21

**Authors:** Sneha Chebrolu, Richard Kazibwe, Elsayed Z. Soliman

**Affiliations:** ^1^ Department of Internal Medicine Wake Forest School of Medicine Winston‐Salem North Carolina USA; ^2^ Section on Cardiovascular Medicine, Department of Medicine, Epidemiological Cardiology Research Center (EPICARE) Wake Forest School of Medicine Winston‐Salem North Carolina USA

**Keywords:** cardiovascular mortality, poverty income ratio, social determinants of health, subclinical myocardial injury

## Abstract

**Introduction:**

Both low family income and subclinical myocardial injury (SCMI) are risk factors for cardiovascular disease (CVD) mortality. However, the impact of their joint association on CVD mortality is unclear.

**Methods:**

This analysis from the third National Health and Nutrition Examination Survey included 6805 participants (age 59.1 ± 13.4 years, 52.3% women, and 49.8% White) free of CVD at baseline. Family income was assessed using the poverty‐income ratio (PIR) and categorized into low (PIR < 1), middle (PIR = 1–4), and high (PIR > 4) income. A validated ECG‐based cardiac infarction injury score (CIIS) ≥ 10 was considered positive for SCMI. CVD mortality was determined using the National Death Index. Cox‐proportional hazard analysis was used to evaluate the associations of family income and SCMI, separately and jointly, with CVD mortality.

**Results:**

A total of 1782 (26.2%) participants had SCMI at baseline. During a median follow‐up of 18.2 years, 856 (12.6%) events of CVD mortality occurred. In separate multivariable Cox models, SCMI (vs. no SCMI) and middle‐ and low‐income (vs. high‐income) were each associated with a higher risk of CVD mortality (HR [95% CI]: 1.34 [1.16–1.54], 1.44 [1.16–1.78], and 1.59 [1.22–2.07], respectively). Compared to high‐income participants without SCMI, those with low‐income and SCMI had an increased risk of CVD mortality (HR [95% CI]: 2.17 [1.53–3.08]). The multiplicative interaction between PIR and SCMI was not significant (*p* = 0.054).

**Conclusion:**

Lower family income and SCMI are associated with CVD mortality, and their concomitant presence is associated with the highest risk. Family income and SCMI may help in the individualized assessment of CVD risk.

## Graphical abstract

This study evaluated the joint associations of family income and SCMI with CVD mortality in participants from NHANES III. We found that lower family income and SCMI are associated with CVD mortality; their concomitant presence is associated with the highest risk. Family income and SCMI may help assess CVD risk.

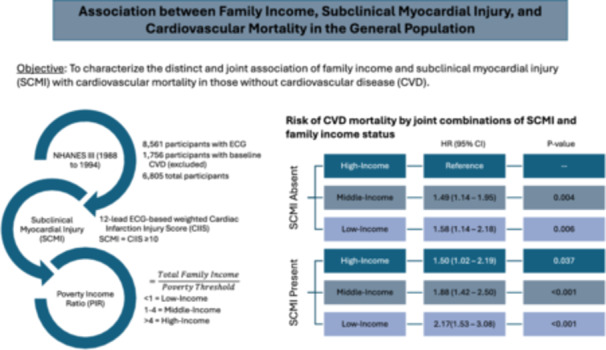

**Abbreviations**
CIIS: cardiac infarction injury score
CVD: cardiovascular disease
HR: hazard ratio
NHANES: The National Health and Nutrition Examination Survey
PIR: poverty‐income ratio
SD: standard deviation
SES: socioeconomic status

## 1 Introduction

Cardiovascular disease (CVD) is a leading cause of death in the United States, with low socioeconomic status (SES) being a recognized contributor to CVD‐related morbidity and mortality [1, 2]. The four measures of SES, income level, educational attainment, employment status, and neighborhood socioeconomic factors, have all been linked to CVD [3]. Family income is an easily quantifiable indicator of SES and can be measured using the poverty‐income ratio (PIR), a standardized income against the federal poverty level [3]. The majority of existing studies on SES and CVD have focused on clinical manifestations of CVD, not subclinical CVD [2, 4]. Identifying risk factors for CVD mortality has become increasingly important given the rising number of deaths attributed to CVD in recent years [5].

The Cardiac Infarction/Injury Score (CIIS) is a validated tool for defining subclinical myocardial injury (SCMI) in population studies [6]. SCMI has been shown to be associated with increased CVD mortality in individuals without a prior history of CVD [7]. Therefore, we sought to examine the impact of the concomitant presence of SCMI and low family income on the risk of CVD mortality.

## 2 Methods

### 2.1 Study Design and Population

We used data from the third National Health and Nutrition Examination Survey (NHANES III). The data are publicly accessible via the National Center for Health Statistics (NCHS) and can be accessed online [8]. NHANES III is an episodic survey of a noninstitutionalized population in the United States conducted from 1988 to 1994 with the goal of providing information on disease prevalence and risk factors. The design and components of the NHANES III have been previously described [9]. It was approved by the NCHS Research Ethics Review Board and documented consent was obtained from participants.

For the purpose of this analysis, we only included NHANES III participants who underwent electrocardiogram recording (*n* = 8561). We excluded participants with prior myocardial infarction, heart failure, and stroke or with missing key variables. After all exclusions (*n* = 1756), 6805 participants were included in this analysis.

### 2.2 Family Income

The PIR was utilized in this analysis as a measure of SES. As described by the United States Census Bureau, the PIR is calculated by dividing the annual total family income by the family's poverty threshold for that year [10]. Total family income excludes capital gains or losses, noncash benefits, and tax credits. The poverty thresholds for each family size are determined annually while accounting for inflation using the Consumer Price Index for All Urban Consumers. Thus, a PIR less than 1 indicates that the total family income is less than the poverty threshold for that family size. We utilized three subgroups in our analysis: low‐income (PIR < 1), middle‐income (PIR 1–4), and high‐income (PIR > 4), similar to prior studies [11, 12]. This is also consistent with the Affordable Care Act, which provides subsidies to families with income levels between 100% and 400% of the federal poverty level [13].

### 2.3 SCMI

SCMI was ascertained using a 12‐lead ECG‐based CIIS. The CIIS has been validated as a tool that can detect myocardial injury using visual or computerized ECG analysis [6]. CIIS is based on a weighted scoring system that considers several objective electrocardiographic waveform components related to myocardial injury and ischemia, generating a risk‐stratified scoring system. The score is defined by a combination of 11 discrete and 4 continuous features, providing a simple scoring scheme suitable for both visual and computer classification of a standard 12‐lead ECG. These features included measurements of Q, R, and T waves as well as ST segments. Consistent with previous studies, we defined SCMI as CIIS ≥ 10 [7, 14, 15, 16, 17, 18].

### 2.4 Cardiovascular Mortality

CVD mortality was determined using data from the NHANES III linked mortality file with the endpoint identified using ICD‐10 codes 100–178. NHANES III participants were linked to death information in the National Death Index using an algorithm that comprises multiple identifiers, including social security number, sex, and date of birth. CVD mortality data among NHANES III participants were accessible through December 31, 2019.

### 2.5 Other Measurements

Baseline demographic information, including participants' age, sex, race/ethnicity, smoking status, and physical activity, was self‐reported using an in‐home interview. Physical examinations were conducted on study participants; body mass index (BMI) was calculated from height and weight measurements with obesity defined as BMI ≥ 30 kg/m^2^. An average blood pressure (BP) from six separate measurements was reported. Blood samples were collected via venipuncture by a phlebotomist. Various laboratory measurements, such as total cholesterol, triglycerides, and glucose, were analyzed using procedures as reported by the NCHS. Hypertension was defined as either systolic BP ≥ 140 mmHg, diastolic BP ≥ 90 mmHg, or self‐reported use of antihypertensive medications. Diabetes was defined as fasting blood glucose ≥ 126 mg/dL or the use of antidiabetic medications. Hyperlipidemia was defined as total cholesterol ≥ 200 mg/dL, triglycerides ≥ 150 mg/dL, or self‐reported use of cholesterol‐lowering medications.

### 2.6 Statistical Analysis

Continuous variables were reported as mean ± standard deviation (SD) or median (interquartile range [IQR]) and categorical variables were reported as frequency with percentage. The baseline characteristics of the participants were stratified by both family income categories and SCMI status. For continuous variables, a comparison was made using *t*‐test or analysis of variance (ANOVA), or Kruskal–Willis test depending on the normality of distribution. Chi‐square test was used for categorical variables. Multivariable logistic regression analysis was used to explore the association between family income and SCMI at baseline. Model 1 adjusted for age, sex, race/ethnicity, and education level (≥ high school vs. < high school). Model 2 adjusted for covariates in model 1 plus diabetes, hypertension, total cholesterol, BMI, lipid‐lowering medications, smoking (ever smoker vs. never smoker), and physical activity (self‐reported participation in recreational or work‐related physical activities). Odds ratios were reported with the corresponding 95% confidence intervals (CIs). Cardiovascular mortality rates as number of events per 1000 person‐years stratified by family income levels and SCMI were calculated. Multivariable Cox‐proportional hazard models were used to assess the associations of family income categories and SCMI, separately and in combination with CVD mortality. Hazard ratios (HRs) and 95% CIs were reported. Similarly, two models were utilized for this analysis, adjusting for the same covariates as in the aforementioned logistic analysis. Additionally, the multiplicative *p* values for interaction between PIR (as a continuous variable) and SCMI, as well as family income category (as a categorical variable) and SCMI were calculated using Model 2 covariates. Further analysis was conducted comparing Black and non‐Black participants to examine whether the effects of income and SCMI varied by race/ethnicity. All statistical analyses were conducted in SAS version 9.4 (SAS Institute Inc., Cary, NC, USA) with a two‐sided significance level of 0.05.

## 3 Results

A total of 6805 participants were included in the analysis (age 59.1 ± 13.4 years, 52.3% women, and 49.8% White). Table 1 shows the baseline characteristics of the study participants stratified by family income levels and SCMI status. Participants in the low‐income group and those with SCMI had lower rates of educational attainment and a higher prevalence of CVD risk factors compared to participants in the high‐income group and those without SCMI.

**Table 1:** Baseline characteristics of study participants.

Characteristics mean ± SD, median (IQR), or *n* (%)	Family income levels				Subclinical myocardial injury (SCMI)		
	Low (*n* = 1304)	Middle (*n* = 4036)	High (*n* = 1465)	*p* value^a^	Absent (*n* = 5023)	Present (*n* = 1782)	*p* value^b^
Age (years)	59.9 ± 13.8	59.6 ± 13.6	57.1 ± 12.0	< 0.001	57.6 ± 13.1	63.5 ± 13.2	< 0.001
Men	555 (42.6%)	2101 (52.1%)	709 (48.4%)	< 0.001	2338 (46.6%)	908 (51.0%)	0.001
Race/ethnicity (Black)	434 (33.3%)	997 (24.7%)	202 (13.8%)	< 0.001	1184 (23.6%)	449 (25.2%)	0.168
Poverty‐income ratio	0.69 (0.50–0.83)	2.27 (1.52–2.96)	5.30 (4.61–6.14)	< 0.001	2.28 (1.20–3.76)	2.08 (1.14–3.48)	< 0.001
≥ High school education	305 (23.4%)	2230 (55.3%)	1293 (88.3%)	< 0.001	2915 (58.0%)	913 (51.2%)	< 0.001
Diabetes	346 (26.5%)	745 (18.5%)	179 (12.2%)	< 0.001	845 (16.8%)	425 (23.8%)	< 0.001
Ever smoker	699 (53.6%)	2200 (54.5%)	843 (57.5%)	0.043	2676 (53.3%)	1066 (59.8%)	< 0.001
BMI	28.1 ± 5.9	27.7 ± 5.6	27.0 ± 4.9	< 0.001	27.6 ± 5.4	27.8 ± 6.0	0.106
Systolic BP (mmHg)	135.2 ± 29.8	133.3 ± 28.4	129.1 ± 18.1	< 0.001	131.2 ± 28.7	137.0 ± 20.3	< 0.001
Diastolic BP (mmHg)	76.8 ± 25.0	77.1 ± 27.4	76.6 ± 9.5	0.762	77.1 ± 27.5	76.3 ± 10.8	0.240
Total cholesterol (mg/dL)	219 ± 46.8	223.1 ± 44.0	222.6 ± 40.8	0.024	221.2 ± 43.4	225.9 ± 47.5	0.023
Lipid lowering medication	48 (3.7%)	145 (3.6%)	77 (5.6%)	0.017	197 (3.9%)	73 (4.1%)	0.746
Physically active	607 (46.6%)	2795 (69.3%)	1224 (83.6%)	< 0.001	3497 (69.6%)	1129 (63.4%)	< 0.001

Abbreviations: BMI, body mass index; BP, blood pressure; IQR, interquartile range.

^a^ *p* value by ANOVA, Kruskal–Wallis test, or chi‐square.

^b^ *p* value by *t*‐test or chi‐square.

**Figure 1:**
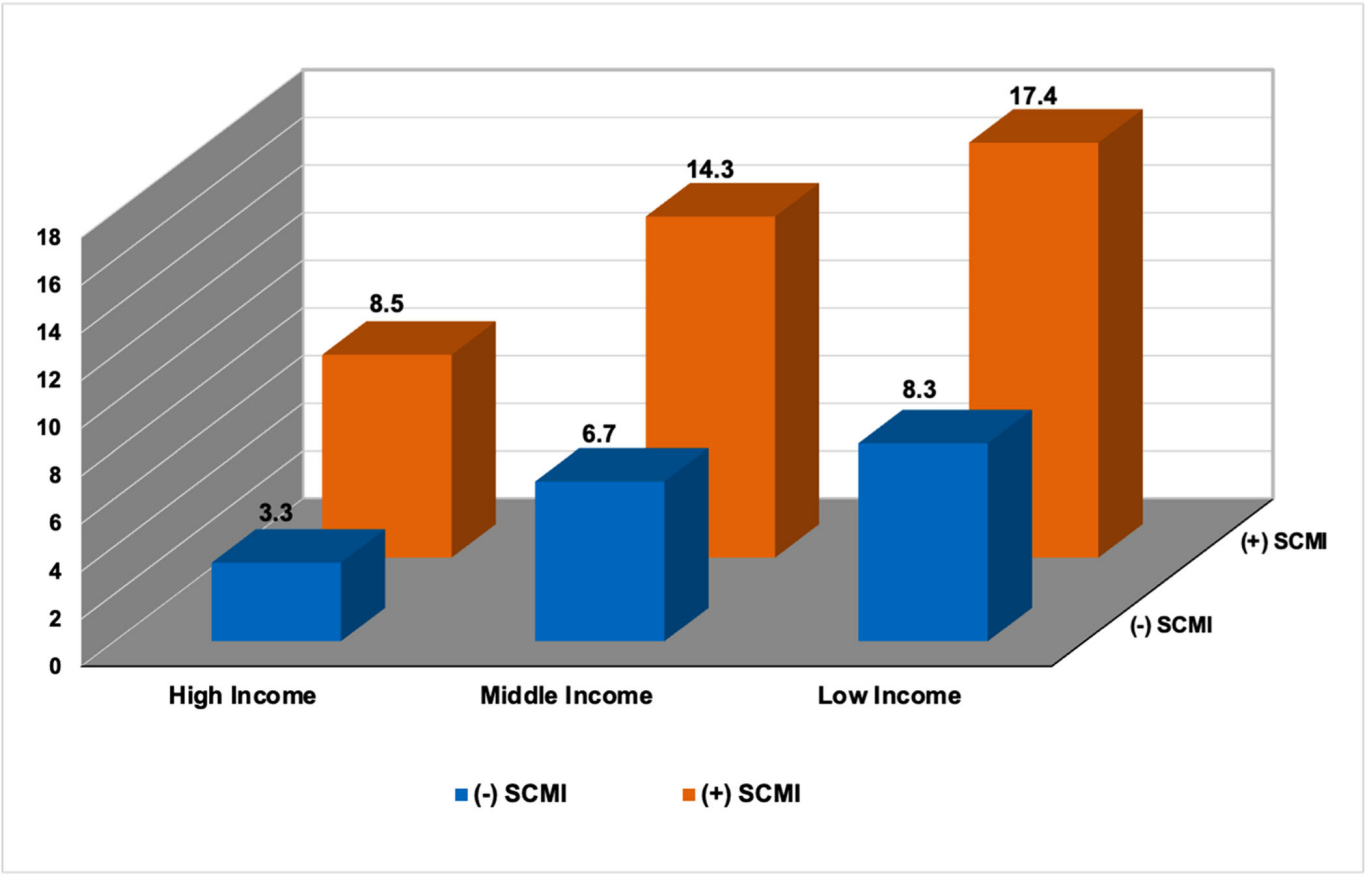
CVD mortality rates stratified by family income level and SCMI status. Mortality rate per 1000 person‐years. SCMI, subclinical myocardial injury.

At baseline, the prevalence of SCMI was 26.6% (1782 participants), and there was no significant association between family income and SCMI (Table 2). During a median follow‐up of 18.2 years (IQR 11.9–20.4 years), 856 CVD deaths occurred. Individuals with SCMI and low‐income had the highest event rate of CVD mortality, while those with neither risk factor had the lowest event rate (Figure 1).

**Table 2:** Baseline association between family income and SCMI.

Family income	Model 1		Model 2	
	OR (95% CI)	*p* value	OR (95% CI)	*p* value
High‐income	Reference	—	Reference	—
Middle‐income	1.16 (0.99–1.34)	0.060	1.12 (0.96–1.30)	0.161
Low‐income	1.17 (0.95–1.43)	0.139	1.06 (0.87–1.31)	0.556

*Note:* Model 1 adjusted for age, sex, race, and education level. Model 2 adjusted for model 1 plus diabetes, hypertension, total cholesterol, body mass index, lipid‐lowering medications, smoking, and physical activity.

Abbreviations: CI, confidence interval; OR, odds ratio.

In a multivariable Cox‐proportional hazards model adjusted for sociodemographics and CVD risk factors, lower and middle family income levels (HR [95% CI]: 1.44 [1.16–1.78] and 1.59 [1.22–2.07], respectively) were associated with increased risk of CVD mortality, compared to high‐income levels. In a similar model, the presence versus absence of SCMI was associated with an increased risk of CVD mortality (HR [95% CI]: 1.34 [1.16–1.54]) (Table 3). The *p* values for multiplicative interaction between PIR or family income category and SCMI were 0.054 and 0.680, respectively.

**Table 3:** Separate association of family income and SCMI with CVD mortality.

Income and SCMI status		Events/1000 person‐years	Model 1		Model 2	
			HR (95% CI)	*p* value	HR (95% CI)	*p* value
Income status	High‐income	4.5	Reference	—	Reference	—
	Middle‐income	8.5	1.60 (1.30–1.96)	< 0.001	1.44 (1.16–1.78)	< 0.001
	Low‐income	10.4	1.95 (1.52–2.50)	< 0.001	1.59 (1.22–2.07)	< 0.001
SCMI	Absent	6.2	Reference	—	Reference	—
	Present	13.6	1.50 (1.30–1.72)	< 0.001	1.34 (1.16–1.54)	< 0.001

*Note:* Model 1 adjusted for age, sex, race, and education level. Model 2 adjusted for model 1 plus diabetes, hypertension, total cholesterol, body mass index, lipid‐lowering medications, smoking, and physical activity.

Abbreviations: CVD, cardiovascular disease; SCMI, subclinical myocardial injury.

Table 4 shows results from a subgroup analysis of income levels and SCMI status. Compared to the high‐income participants without SCMI, those with low‐income with SCMI had an increased risk of CVD mortality (HR [95% CI]: 2.17 [1.53–3.08]).

**Table 4:** Risk of CVD mortality by joint combinations of SCMI and family income status.

Categories of family income and SCMI status	Events/1000 person‐years	Model 1		Model 2	
		HR (95% CI)	*p* value	HR (95% CI)	*p* value
SCMI absent + high‐income	3.3	Ref.	—	Ref.	—
SCMI absent + middle‐income	6.7	1.56 (1.19–2.03)	0.001	1.49 (1.14–1.95)	0.004
SCMI absent + low‐income	8.3	1.81 (1.31–2.49)	< 0.001	1.58 (1.14–2.18)	0.006
SCMI present + high‐income	8.5	1.64 (1.12–2.39)	0.010	1.50 (1.02–2.19)	0.037
SCMI present + middle‐income	14.3	2.22 (1.68–2.94)	< 0.001	1.88 (1.42–2.50)	< 0.001
SCMI present + low‐income	17.4	2.59 (1.83–3.66)	< 0.001	2.17 (1.53–3.08)	< 0.001

*Note:* Model 1 adjusted for age, sex, race, and education level. Model 2 adjusted for model 1 plus diabetes, hypertension, total cholesterol, body mass index, lipid‐lowering medications, smoking, and physical activity.

Abbreviations: CVD, cardiovascular disease; SCMI, subclinical myocardial injury.

The separate association of family income and SCMI with CVD mortality, when stratified by race/ethnicity, was not significant (Table S1).

## 4 Discussion

In this analysis from the NHANES III, we examined the interrelationship between family income, SCMI, and CVD mortality. In separate models, we found that low family income and presence of SCMI were associated with an increased risk of CVD mortality. We also found that concomitant presence of those two risk factors was associated with a higher risk of CVD risk compared to the presence of either risk factors alone. These findings suggest that family income status and SCMI may help in the individualized assessment of CVD risk.

SES is multifaceted and can be difficult to assess as it includes multiple variables such as education, income, housing, and food security [2, 19]. SES has been associated with premature mortality (death before age 70) at a level that is comparable to traditional CVD risk factors making it an important measure to include when evaluating health outcomes [20]. The use of SES can help to characterize the ability of patients to utilize the healthcare system. The association of lower SES with CVD and mortality has been established in both national US and global populations [21, 22, 23]. Within the NHANES population, the association of SES with CVD and co‐morbidities (diabetes mellitus, hypertension, hyperlipidemia, smoking, and stroke), as well as all‐cause and cardiac mortality has been demonstrated [3, 11, 12, 24, 25, 26, 27, 28]. These findings are in agreement with our observation of the increased risk of CVD mortality associated with low family income. We specifically focused on income as a measure of SES as income directly limits the ability to access healthcare. PIR is indicative of household income; this is a more robust way to quantify SES than individual income since multiple individuals in a household may share resources. An analysis of SES factors suggested that economic markers like income were better at determining association with mortality than social components such as education [29].

There are limited studies on the associations between SES and SCMI. A cross‐sectional study using high‐sensitivity cardiac troponin T to evaluate SCMI found that lower SES, defined by both income and educational level, was associated with increased levels of troponin [30]. However, we could not find a significant association between family income and SCMI in NHANES III. Further, there was no significant interaction between PIR (or family income category) and SCMI on CVD mortality. These results suggest that SES, specifically family income in this analysis, and SCMI impact CVD mortality using independent pathways. We also found that race/ethnicity did not change the prognostic value of family income or SCMI.

The benefit of CIIS is that it is easily obtained from a cost‐effective ECG while providing profound information that can direct future management. For lower income groups that have challenges in obtaining cardiovascular health care, integrating affordable tools like ECG and CIIS into clinical practice would be particularly beneficial.

The strength of our study is that it utilized a large national, multiracial sample with a long follow‐up time. Our study has some limitations. First, while we have made efforts to account for potential confounding variables, we acknowledge the possibility of residual confounding. For instance, undiagnosed CVD among those we included in the analysis cannot be entirely ruled out. Second, family income level, which was used as a measure for SES, was self‐reported, introducing the potential for recall bias and misclassification. Third, we did not account for changes in family income over time due to the unavailability of this data. While the long follow‐up period may have allowed for analysis of income fluctuations and the potential impact on outcomes, this limitation provides an opportunity for future research to investigate the effects of income variation.

## 5 Conclusions

In individuals without CVD, both low‐income and SCMI are independently associated with increased risk for CVD mortality, and the highest risk was observed in those where low‐income and SCMI coexist. Further research is warranted to examine the utility of SCMI as a predictor of CVD mortality within the context of socioeconomic factors.

## Ethics Statement

NHANES III was approved by the National Center for Health Statistics Research Ethics Review Board.

## Consent

Documented consent was obtained from participants as part of NHANES III.

## Conflicts of Interest

The authors declare no conflicts of interest.

## Supporting information

Supporting information.

## Data Availability

The data that support the findings of this study are openly available in NHANES III at https://wwwn.cdc.gov/nchs/nhanes/default.aspx.
