# Supplementary material for: Association Between Family Income, Subclinical Myocardial Injury, and Cardiovascular Mortality in the General Population
Source: Clin Cardiol. 2024 Oct 21;47(10):e70036. doi: 10.1002/clc.70036 (PMC11491868; doi:10.1002/clc.70036)
Supplement: Supplementary file 1 — Supporting information. [file CLC-47-e70036-s001.docx]

**Supplemental Table 1: Separate association of family income and SCMI with CVD mortality stratified by race/ethnicity**

|  | | **Participants (n)**  **/Events (1000 person-years)** | **HR**  **(95% CI)** | **Interaction**  **P-value** |
| --- | --- | --- | --- | --- |
| **Income Status** | | | | |
| **Black** | High-Income | 202/1.1 | Ref. | 0.276 |
|  | Middle-Income | 997/6.6 | 3.67(1.33 – 10.08) |  |
|  | Low-Income | 434/10.8 | 4.30(1.52 – 12.18) |  |
| **Non-Black** | High-Income | 1263/5.0 | Ref. |  |
|  | Middle-Income | 3039/9.1 | 1.27(1.02 – 1.58) |  |
|  | Low-Income | 870/10.2 | 1.26(0.95 – 1.67) |  |
| **SCMI Status** | | | | |
| **Black** | SCMI Absent | 1184/5.6 | Ref. | 0.381 |
|  | SCMI Present | 449/10.8 | 1.10 (0.81 – 1.50) |  |
| **Non-Black** | SCMI Absent | 3839/6.4 | Ref. |  |
|  | SCMI Present | 1333/14.6 | 1.40(1.20 – 1.64) |  |
| *Model adjusted for age, sex, education level, diabetes, hypertension, total cholesterol, body mass index, lipid lowering medications, smoking, and physical activity* | | | | |
